# Identification of Continuous Human B-Cell Epitopes in the Envelope Glycoprotein of Dengue Virus Type 3 (DENV-3)

**DOI:** 10.1371/journal.pone.0007425

**Published:** 2009-10-13

**Authors:** Andréa N. M. Rangel da Silva, Eduardo J. M. Nascimento, Marli Tenório Cordeiro, Laura H. V. G. Gil, Frederico G. C. Abath, Silvia M. L. Montenegro, Ernesto T. A. Marques

**Affiliations:** 1 Virology and Experimental Therapy Laboratory, Aggeu Magalhães Research Center, Fiocruz, Recife, Pernambuco, Brazil; 2 Department of Immunology, Aggeu Magalhães Research Center, Fiocruz, Recife, Pernambuco, Brazil; 3 Central Laboratory of Public Health, Secretaria de Saúde do Estado de Pernambuco, Brazil; 4 The Johns Hopkins School of Medicine, Department of Medicine, Division of Infectious Diseases, Baltimore, Maryland, United States of America; 5 Department of Pharmacology and Molecular Sciences, The Johns Hopkins School of Medicine, Baltimore, Maryland, United States of America; New York University School of Medicine, United States of America

## Abstract

**Background:**

Dengue virus infection is a growing global public health concern in tropical and subtropical regions of the world. Dengue vaccine development has been hampered by concerns that cross-reactive immunological memory elicited by a candidate vaccine could increase the risk of development of more severe clinical forms. One possible strategy to reduce risks associated with a dengue vaccine is the development of a vaccine composed of selected critical epitopes of each of the serotypes.

**Methodology/Principal Findings:**

Synthetic peptides were used to identify B-cell epitopes in the envelope (E) glycoprotein of dengue virus type 3 (DENV-3). Eleven linear, immunodominant epitopes distributed in five regions at amino acid (aa) positions: 51–65, 71–90, 131–170, 196–210 and 246–260 were identified by employing an *enzyme- linked immunosorbent assay* (ELISA), using a pool of human sera from dengue type 3 infected individuals. Peptides 11 (aa51–65), 27 and 28 (aa131–150) also reacted with dengue 1 (DENV-1) and dengue 2 (DENV-2) patient sera as analyzed through the ROC curves generated for each peptide by ELISA and might have serotype specific diagnostic potential. Mice immunized against each one of the five immunogenic regions showed epitopes 51–65, 131–170, 196–210 and 246–260 elicited the highest antibody response and epitopes131–170, 196–210 and 246–260, elicited IFN-γ production and T CD4+ cell response, as evaluated by ELISA and ELISPOT assays respectively.

**Conclusions/Significance:**

*O*ur study identified several useful immunodominant IgG-specific epitopes on the envelope of DENV-3. They are important tools for understanding the mechanisms involved in antibody dependent enhancement and immunity. If proven protective and safe, in conjunction with others well-documented epitopes, they might be included into a candidate epitope-based vaccine.

## Introduction

Dengue infections occur in the most of tropical and subtropical areas of the world, and are considered as one of the most important re-emerging disease [1]. More than 2.5 billion people live in areas at risk of dengue infection and more than 100 countries have endemic dengue transmission [2]. Dengue virus is a member of the family *Flaviviridae*, genus *Flavivirus* with four antigenically distinct serotypes (DENV-1 to DENV-4). Dengue is a single-stranded, positive-sense, RNA virus with a genome of approximately 11 kb and it encodes three structural (Capsid, C; pre-membrane, prM and envelope, E) and seven nonstructural (NS) proteins (NS1, NS2a, NS2b, NS3, NS4a, NS4b and NS5) [3]. The E glycoprotein is exposed on the surface of the dengue virion particle and is responsible for virus attachment, virus-specific membrane fusion and virus assembly [4]. The E glycoprotein is the most relevant B-cell antigen, conferring protective immune responses by eliciting neutralizing antibodies and also no-neutralizing antibodies, which can be involved in enhancement of virus infection. The *flavivirus* E-glycoprotein contains three structural and functional domains: domains I and III contain predominately subcomplex and type-specific epitopes, whereas domain II contains the major flavivirus group and subgroup cross-reactive epitopes [5]. There is no available antiviral therapy to treat dengue infections and control of dengue virus by vaccination has proved elusive [6]. One ideal strategy to develop an effective and safe dengue vaccine is the design of an epitope-based vaccine containing selected immunogenic targets correlated with protection and devoid the ones associated with pathogenic reactions. Epitopes in the E-glycoprotein [7]–[10], NS1 [11]–[13], NS4a and capsid [3] for DENV-1 and DENV-2 have been mapped. However, only few linear immunogenic determinants on the surface of the virion of each dengue serotype have been identified and a detailed map of the dengue linear B-cell epitopes still need to be determined [14]. Most known neutralizing epitopes are conformational, however continuous amino acid sequences corresponding to antigenic determinants of the proteins that elicit antibodies in dengue-infected patients are more suitable for protein engineering and vaccine development. In particular, not much information is available in the literature regarding the B-cell epitopes in the proteins of DENV-3 [15]. In the present study, we have screened for B cell epitopes in the E glycoprotein of DENV-3 recognized by serum of humans infected with DENV-3 and have identified several immunodominant IgG-specific epitopes.

## Materials and Methods

### Peptide synthesis

A total of ninety-five peptides (each with 15-mers, overlap of 10) were synthesized by Synpep Laboratory, California-USA. They were produced covering the 490 amino acids (aa) of the E protein sequence deducted from the genome of a Dengue 3 isolated from Brazil [16]. For convenience, this sequence will be cited as DENV-3seq. At first, these peptides were used to perform first screening with pool of patient sera. For screening using different groups of patients with dengue fever (DF), dengue hemorrhagic fever (DHF) and patients with DENV-1 or DENV-2 infections, the synthetic peptides were used in a purest form (Schafer Laboratory, Copenhagen-DK), covering all amino acids of the envelope protein. All synthetic peptides used were similar and the only difference between them was the Laboratory (Synpep or Schafer) where they were synthesized.

### Hydropathicity, surface accessibility and antigenicity analysis

DENV-3seq was analyzed using Lasergene software (DNASTAR Inc.) and putative hydrophobic and hydrophilic regions along the length predicted using Kyte and Doolittle method [17]. Surface accessibility and antigenicity analysis were done using Emini [18] and [19] prediction methods respectively, both available at “Immune Epitope Database and Analysis Resource” home page (www.immunoepitope.com). Other surface accessibility prediction was done with DSSP program [20].

### Modeling DENV-3seq E-glycoprotein

The homology modeling of DENV-3seq structure was done using the First Approach Mode under the Swiss Model program (www.swissmodel.expasy.org//SWISS-MODEL.html) [21] and the Deep Viewer/Swiss PBD Viewer program was required for visualization (www.expasy.org/spdbv/) [22].

### Dengue patient sera

For the first screening using synthetic peptides, sera from eight patients that were positive for anti-dengue serotype 3 antibodies were pooled together and used as positive control. The diagnostic of these patients were performed for IgM (*anti-dengue IgM-capture ELISA, Bio-manguinhos, Fundação Oswaldo Cruz, Brazil)* and IgG antibodies (*anti-dengue IgG- ELISA, PanBio, Pty., LTd., Brisbane, Australia and* in house *indirect anti-dengue IgG-ELISA*). As negative control, peptides were screened with a serum pool from twenty-one individuals that were determined to be negative for DENV-3, and also for DENV-1 and DENV-2 by multiple serological detection methods. The Virology Branch of the Public Health Central Laboratory of Pernambuco (LACEN-PE) provided pooled sera for the standard control (negative and positive controls). The peptides mapped in this first screening were used for testing individually sera from thirty-two positive patients and seventeen dengue naive individuals, in order to investigate the capacity of the epitopes in discriminate between serum DENV-3 patients from non-dengue patients. The patient serum samples were obtained from a dengue cohort developed at the Virology and Experimental Therapy Laboratory (LAVITE), of the Aggeu Magalhães Research Center Recife, Brazil described elsewhere [23]. The sera for DENV-3 were identified as positive through conventional laboratory assays such as viral isolation, RT-PCR, IgM and IgG antibodies detection. The normal sera were defined as those who presented to be negative for IgM and IgG anti-dengue antibodies. To investigate the power of the first mapped peptides in discriminate the infections of DENV-3 from that of other dengue serotypes, sera from twenty patients with defined DENV-1 or DENV-2 infections, diagnosed as positive during 1999 Brazilian's dengue outbreak were used, provided by Virology Branch of the Public Health Central Laboratory of Pernambuco (LACEN-PE).

### Ethical considerations

Written consent to participate in the study was obtained from each patient (or the patient's guardian) after a full explanation of the study was provided. All data were handled confidentially and anonymously. This study was reviewed and approved by the ethics committee of the Brazilian Ministry of Health (N° 4909 CONEP) and The Johns Hopkins University School of Medicine internal review board (# 03-08-27-01).

### Immunogenicity of human DENV-3 envelope B cell epitopes on Balb/c mice

Balb/c mice were purchased from Jackson Laboratories (Maryland, USA) and maintained in a helicobacter-negative environment at the Johns Hopkins School of Medicine Animal Facility. Mice were divided in six groups (two animals per group), whereby five groups represented each of the five dengue E-glycoprotein epitope regions found on human cohort, whereas the sixth group was the negative control. The animals were immunized subcutaneously (base of the tail) twice, three weeks apart, with total amount of peptide of 50 µg, emulsified in TiterMax, per epitope region. When the epitope region was composed of two or more peptides, they were polled together so that the final amount of peptides injected was 50 µg. The epitope regions and the peptide sequences analyzed are described below: (a) 51–65 (TQLATLRKLCIEGKI); (b) 71–90 [DSRCPTQGEAVLPEE (71–85) and TQGEAVLPEEQDPNY (76–90)]; (c) 131–170 [QYENLKYTVIITVHT (131–145), KYTVIITVHTGDQHQ (136–150), ITVHTGDQHQVGNET (141–155), GDQHQVGNETQGVTA (146–160), VGNETQGVTAEITPQ (151–165) and QGVTAEITPQASTTE (156–170)]; (d) 196–210 (LLTMKNKAWMVHRQW); and (e) 246–260 (PEVVVLGSQEGAMHT). The negative control animals were immunized with aqueous solution of Titermax. The sera of immunized mice and controls were collected before immunization, three weeks after priming and two weeks after boosting for humoral and cellular immune response analysis.

### ELISA (enzyme linked immunosorbent assay) for peptide epitope identification

ELISA was optimized using Synpep linear synthetic peptides from E-glycoprotein. Briefly, each microtiter well (Immulon II, Dynatech Laboratories Inc., VA-USA) was coated overnight at 4°C with 20 µg/ml in 100 µl/well of synthetic peptides in 0.2 M carbonate buffer (CB), pH 9.6. Wells were then blocked with 200 µl of 5% dry skimmed milk in CB for 2 h at room temperature (RT). The wells were washed in 0.05% Tween 20 (Reagen, RJ, Brazil) in phosphate buffered saline (PBS), pH 7.2, and then 1∶50 human sera diluted in 10% dry skimmed milk in PBS was added and incubated for 1 h at 37°C. Another wash followed with 0.05% Tween 20 in PBS and wells were incubated for 2 h at RT with 100 µl of 1∶1,000 peroxidase mouse anti-human IgG monoclonal antibody (Zymed Laboratories Inc., CA-USA), diluted in 10% dry skimmed milk in PBS. Finally, the enzyme activity was detected with the addition of 100 µl of tetramethylbenzydine (TMB) substrate solution at 30 min in RT. Optical densities (ODs) were measured at 450 nm in the microtiter plate reader (Bio-Rad Benchmark Plus). As an internal positive reference control, total cellular extract of DENV-3 infected cells was used as the capture antigen [24].

### ELISA for assessment of immunogenicity of envelope B cell epitopes on Balb/c mice

For epitope validation on sera collected from peptide-immunized Balb/c, Nunc microplates were coated with 100 µg/ml of peptides (100 ul/well) diluted in carbonate/bicarbonate buffer pH 9.6 and incubated overnight at 4°C. After washing with PBS pH 7.2 containing 0.05% Tween 20 (PBS-T) the plates were blocked with 5% BSA (bovine serum albumin, Sigma) in PBS pH 7.2 for 1 h at 37°C. Then, the plates were washed and incubated 2 h at 37°C with serially diluted sera (from 1∶100 to 1∶10,000,000) in dilution buffer (BSA 0.1% in PBS pH 7.2). The samples were analyzed in duplicate. As a negative control (NC), the same diluted sera were incubated in uncoated wells. The plates were washed and incubated for 45 minutes at 37°C with a HRP (horseradish peroxidase) -conjugated goat anti-Mouse IgG (Fab)2 (Jackson Immunoresearch) diluted 1,000-fold in dilution buffer. Then, the plates were washed, developed with TMB (BD Biosciences) and read at 450 nm in a microplate reader (Safari2, Tecan).

### ELISPOT (enzyme linked immunosorbent spot) assay

The IFN-γ ELISPOT assay was performed by use of the ELISPOT set (BD-Biosciences Pharmingen, San Diego, CA) as described by the manufacturer. Briefly, PVDF (polyvinylidene fluoride) plates were coated with mAb (monoclonal antibody) to mouse IFN-γ (5 µg/mL) diluted in PBS and incubated at 4°C overnight. The plates were washed and blocked with RPMI 1640 containing 10% FBS (fetal bovine serum) for 2 hs at room temperature (RT). Splenocytes were harvested from each mice group as described above, being plated in three different ways: (i) as total cells; (ii) as CD4+ T cell depleted splenocytes (CD8+ T cell enriched sample); and (iii) CD8+ T cell depleted splenocytes (CD4+ T cell enriched sample). CD4+ and CD8+ T cell depletions were performed by using immune-magnetic beads coated with antibodies (Abs) to CD4+ and CD8+, respectively, and depletion columns according to manufacturer's protocol (Miltenyi Biotec). One hundred µL of cell suspension, containing in a range of 0.3×10^6^ to 1×10^6^ cells were added to wells containing 100 µL of peptides representing each epitope region identified in humans at a final concentration of 10 µg/mL. Concanavalin A (Sigma-Aldrich, St. Louis, MO), 5 µg/ml, and culture medium were used as positive and negative controls, respectively. Plates were incubated overnight at 37°C, in 5% CO_2_, washed with PBS-T (PBS containing 0.05% Tween 20), and incubated with biotinylated anti-mouse IFN-γ (2 µg/ml) for 2 h at RT. After washing step, the plates were incubated with 100-fold diluted streptavidin-HRP for 1 h at RT, followed by washing and incubation with 3-amino-9-ethylcarbazole substrate for 20 min at RT. The reaction was stopped with distilled water. After the plates were air-dried, the spots were counted by using a CTL analyzer, with the ImmunoSpot® software. The average number of spot forming cells (SFC) was normalized to 1×10^6^ splenocytes. The results were considered significant when the average experimental SFC less 2 standard deviation (SD) was greater than the average of the background plus 2 SD and the average of the experimental peptides was greater than 10 SFCs per 10^6^ cells.

### Mapping B cell epitopes

For the purpose of first assigning the peptide determinants reactive with patient serum, all ninety five peptides provided by Synpep were tested for the positive and negative pool of sera as described above. The mean OD obtained using negative sera pool plus three times standard deviation was used as the cut-off value. The most reactive peptides found at this first screening were tested with the individual patients sera and the results were expressed in ELISA arbitrary unity form (EAU), consisting in the ratio between the OD found for each tested sample and the OD found for the positive reference control (a total cellular extract of DENV-3 infected cells) [24] in the same plate, in order to minimize the variability of positive and negative controls, as well as of the samples in each assay.

### Statistical Analysis

Medcalc version 8.2 was used for (receiver operating characteristic) ROC analysis, plotting and determination of the cut-off. ROC plot displayed sensitivity versus 1-specificity, such that the areas under the curve (AUCs) generated varied from 0.5 to 1.0, with higher values indicating increased discriminatory ability. When the variable under study cannot distinguish between two groups, the area will be equal to 0.5 (the ROC curve will coincide with the diagonal). The cut-off was selected to yield the highest accuracy to discriminate DENV-3 and non-dengue infections or other serotypes infections (minimal false negative and false positive results). Comparisons of AUCs were performed using non-parametric assumptions. Sensitivity, specificity using a confidence interval of 95% for each one of the eleven peptides were calculated and the differences were considered significant if *P*<0.05.

## Results

### Identification of E-glycoprotein epitopes

**Figure 1:**
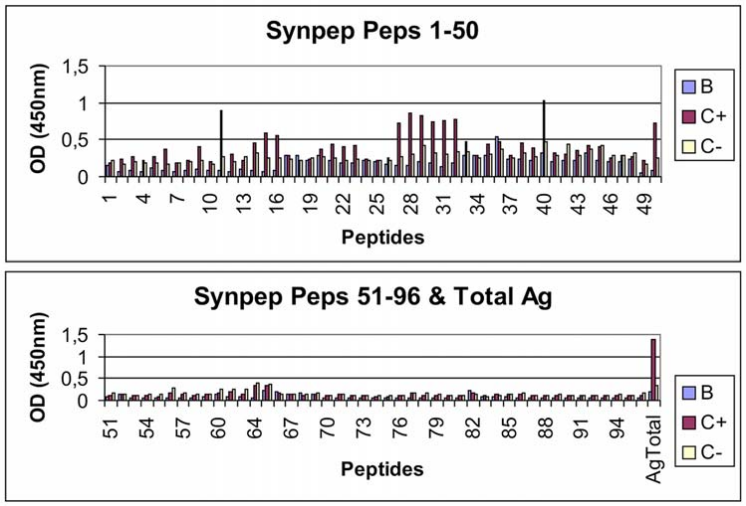
IgG-specific activities of synthetic peptides of DENV-3 E-glycoprotein, using human sera. The horizontal axis denotes the peptide number and the vertical axis denotes the absorbance measured at 450 nm. The cut-off absorbance used to identify reactive epitopes is shown by the red line. Ag, antigen; Peps, peptides; B, blank; C+, positive control; C-, negative control.

The ELISA results showed that the human dengue serum reacted with eleven peptides along the entire length of the E-glycoprotein molecule. These results are shown in the main panel of Figure 1. These peptides are distributed in five continuous regions at amino acid positions 51–65, 71–90, 131–170, 196–210 and 246–260. The sequences of these peptides and their relative epitope activity are summarized in Table 1.

**Table 1:** Immunoreactive DENV- 3 E-glycoprotein peptides.

Peptide #	Peptide sequence (aa residue #s)	ELISA(OD_450_)Human sera
11	TQLATLRKLCIEGKI (51–65)	0,90
15	DSRCPTQGEAVLPEE (71–85)	0,59
16	TQGEAVLPEEQDPNY (76–90)	0,55
27	QYENLKYTVIITVHT (131–145)	0,72
28	KYTVIITVHTGDQHQ (136–150)	0,87
29	ITVHTGDQHQVGNET (141–155)	0,82
30	GDQHQVGNETQGVTA (146–160)	0,75
31	VGNETQGVTAEITPQ (151–165)	0,76
32	QGVTAEITPQASTTE (156–170)	0,78
40	LLTMKNKAWMVHRQW (196–210)	1,03
50	PEVVVLGSQEGAMHT (246–260)	0,72

**ELISA**; enzyme linked immunosorbent assay.

**OD**; optical densities.

### Analyzing hydropathicity, surface accessibility and antigenicity profiles

Parameters such as hydrophilicity, flexibility, accessibility, turns and antigenic propensity of polypeptides chain have been correlated with the location of continuous epitopes. This has led to a search for empirical rules that would allow the position of continuous epitopes to be predicted from certain features of the protein sequence. The evaluation of DENV-3seq, showed that all eleven peptides are in hydrophilic areas according to Kyte and Doolittle hydropathy profiles suggesting that these regions are exposed at the surface of the E protein. However, in the surface accessibility prediction just three peptides from eleven, those including residues VQYENLK (aa 130–136) and TGDQHQVGNETQ (aa 145–156), appearing to be really exposed in the protein surface. In the antigenicity prediction was found that six peptides, those spanning the residues TQLATLRKLCIEG (aa 51–63), QGEAVLPE (aa 77–84), EGKVVQYENLKYTVIITVHTG (aa 126–146) and KKPEVVVLGS (aa 244–253) were considered the most antigenic.

### Modeling DENV-3seq

**Figure 2:**
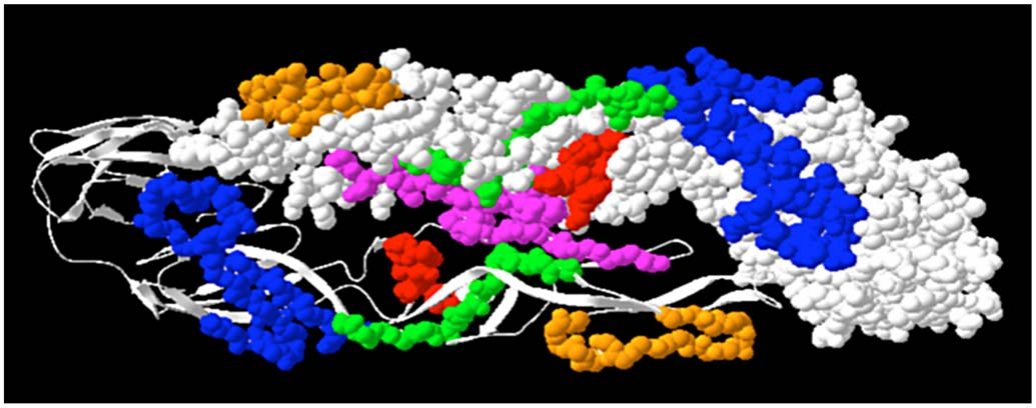
Top view of the homology model of the DENV-3seq E-glycoprotein dimer and location of the eleven mapped peptides. Peptides are represented in colorful space-filling: peptide 11 in green, peptides 15 and 16 in orange, peptides 27, 28, 29, 30, 31 e 32 in blue, 40 in red and 50 in violet. For illustrative reasons one monomer is represented as space-filling and the other as ribbons.

The amino acid sequence of the DENV-3seq was sent to the First Approach Mode under the Swiss Model server [21]. The Swiss Model server returned a monomer of the DENV-3 seq structure based on homology modeling with the published DENV-3 crystal structure (1UZG.pdb, 95.9% of sequence identity) [6] deposited in the Protein Data Bank- PDB [25]. Since the DENV-3 E-glycoprotein molecule consists of a dimer, just for illustrative purposes, a dimer of DENV-3seq structure was built by superimposing the two monomers on the dimer consisting the structure 1UZG.pdb using Deep View/Swiss-PdbViewer program version 3.7/SP5 [22]. The homology model based on the structure 1UZG.pdb also lacks 101 amino acids (positions 558 to 659) which have not been crystallized within the published DENV-3. Figure 2 is shown for illustrative purposes only and a better refinement to predict the exact dimer formation and carbohydrate composition should be taken into consideration. The Swiss Model server is only based on the amino acid sequence of the protein, therefore correct carbohydrate orientation would have been difficult to predict without further analysis. The dimer in Figure 2, shows the position of the eleven mapped peptides in the E-glycoprotein structure, in different colors.

### Analyzing the reactivity profile of eleven mapped peptides to discriminate the infections between DENV-3, other dengue serotypes (DENV-1 and DENV-2) and non-dengue patients

**Figure 3:**
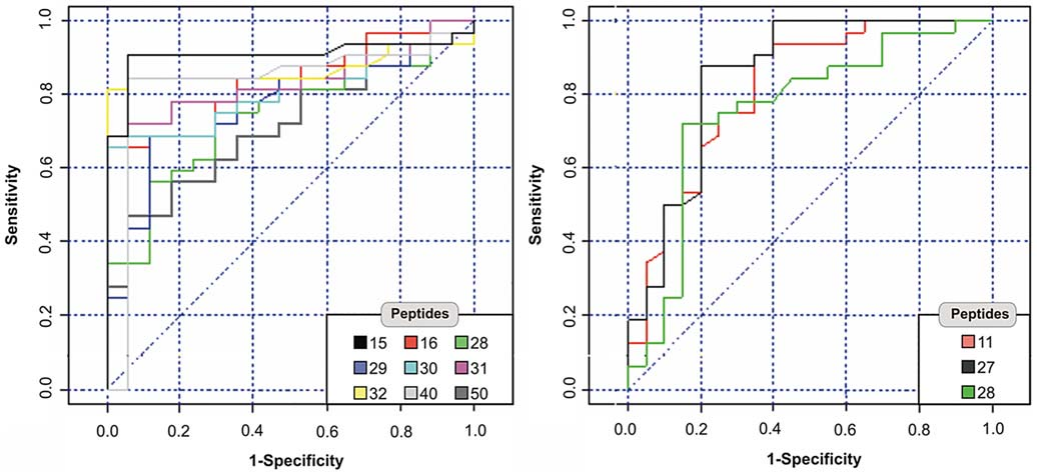
ROC curves generated by Medcalc Software, 8.2 version. (A) Peptides 15, 16, 28, 29, 30, 31, 32, 40, 50 and its power to discriminate between DENV-3 infections and non dengue. (B) Peptides 11, 27, 28 and its power to differentiate DENV-3 infection from dengue infection of other serotypes.

The results were analyzed through the ROC curves generated for each peptide. The ability of the antibody response to discriminate the serum from an infected person from an individual that was not exposed to the dengue virus was evaluated using ROC curve analysis, which can also be used to compare the diagnostic performance of two or more tests. ROC curve is a graphical plot of the sensitivity versus (1-specificity) for a binary classifier system as its discrimination threshold is varied. The accuracy can be measured by the area under the curve (AUC). The bigger the area, the greater the performance of the test. If the area is 1.0, you have an ideal test, because it achieves both 100% sensitivity and 100% specificity. If the area is 0.5, then you have a test, which has effectively 50% sensitivity and 50% specificity. The closer the area is to 1.0, the better the test is to discriminate in this case, the infections between DENV-3 and non-dengue patients and between DENV-3 and infections for other dengue serotypes (DENV-1 and DENV-2). AUC significantly different of 0.5 (*P*<0.05) indicates the power of the peptides to discriminate between the studied groups. The peptides indentified in the first screening were used for testing individually sera from thirty-two positive patients and seventeen dengue naive individuals, in order to investigate the capacity of the epitopes in discriminate between serum DENV-3 patients from non-dengue patients. The assays demonstrates that peptides 15, 16, 28, 29, 30, 31, 32, 40 and 50 have some power to discriminate between DENV-3 infections and non dengue (Table 2 and Figure 3A), with likely potential for using in the diagnostic assays for DENV-3 infections, on the contrary of peptides 11 and 27, that did not perform well. To investigate if the epitopes could discriminate the infections of DENV-3 from that of other dengue serotypes, we assayed the sera from twenty patients with defined DENV-1 or DENV-2 infections. The AUC showed that peptides 11, 27 and 28 (Table 3 and Figure 3B) presented significant performances discriminating the dengue serotype. In this case, patients with DENV-1 and 2 serotypes infections answered to peptides 11, 27 and 28 with higher OD levels than patients with DENV-3.

**Table 2:** Performance of E protein peptides to discriminate dengue infections: DENV-3 infections and non-dengue.

Peptides	CO	Sens.	Spec.	AUC	95% CI	*p value*
11	0,139	59,4	58,8	0,566	0,417–0,707	0,4385
15	0,379	46,9	94,1	0,718	0,571–0,837	0,0030
16	0,372	65,6	94,1	0,835	0,701–0,925	<0,0001
27	0,272	28,1	94,1	0,528	0,381–0,673	0,7428
28	0,231	56,2	88,2	0,738	0,593–0,853	0,0008
29	0,461	68,7	88,2	0,767	0,625–0,876	0,0001
30	0,461	65,6	94,1	0,813	0,676–0,910	<0,0001
31	0,445	68,7	94,1	0,836	0,703–0,926	<0,0001
32	0,419	81,2	94,1	0,869	0,742–0,948	<0,0001
40	0,189	84,4	94,1	0,831	0,697–0,923	<0,0001
50	0,232	90,6	94,1	0,907	0,789–0,971	<0,0001

Sensibility (Sens.), specificity (Spec.), cut-off (CO) and area under curve (AUC) generated for each one of the eleven peptides in the ROC curve. Confidence interval (CI) and *p value* are shown.

**Table 3:** Performance of E protein peptides to discriminate dengue infections: DENV-3 and dengue for other serotypes infections.

Peptides	CO	Sens.	Spec.	AUC	95% CI	*p value*
11	0,27	90,6	65,0	0,816	0,684–0,909	<0,0001
15	0,331	43,7	85,0	0,614	0,469–0,746	0,1634
16	0,826	93,7	20,0	0,530	0,387–0,670	0,7144
27	0,34	87,5	80,0	0,851	0,725–0,934	<0,0001
28	0,329	71,9	85,0	0,759	0,621–0,867	0,0003
29	0,951	90,6	25,0	0,505	0,363–0,646	0,9550
30	0,326	21,9	95,0	0,547	0,403–0,685	0,5732
31	1,197	100,0	20,0	0,536	0,392–0,675	0,6660
32	0,801	62,5	60,0	0,505	0,363–0,646	0,9550
40	0,235	75,0	45,0	0,500	0,358–0,642	1,00
50	0,318	78,1	40,0	0,493	0,325–0,635	0,9326

Sensibility (Sens.), specificity (Spec.), cut-off (CO) and area under curve (AUC) generated for each one of the eleven peptides in the ROC curve. Confidence interval (CI) and *p value* are shown.

### Immunogenicity of Human DENV-3 Envelope B and T cell Epitopes on Balb/c Mice

**Figure 4:**
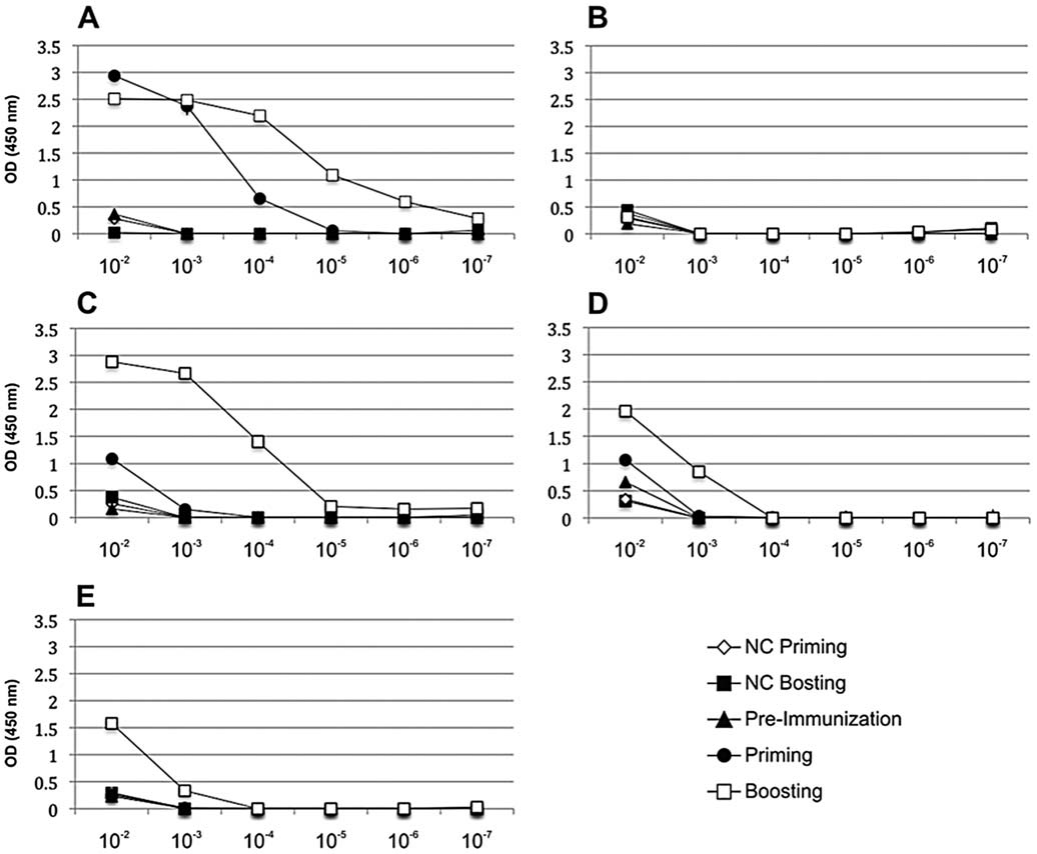
Total IgG production in Balb/c mice elicited by envelope epitopes. (A) 51–65, (B) 71–90, (C) 131–170, (D) 196–210 and (E) 246–260 identified in naturally dengue-infected humans. Balb/c mice were immunized twice with different epitopes and the humoral immune response (pooled sera, n = 2 per group) was assessed through ELISA after priming and boosting in different serum dilutions. NC, negative control.

**Figure 5:**
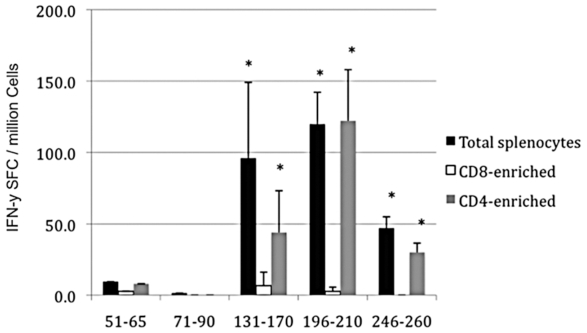
T-cell response through ELISPOT assay for IFN-γ elicited in Balb/c mice immunized with the envelope epitopes identified in human. The T cell response was assessed using total splenocytes harvested from immunized mice as well as from CD8+ T cell enriched splenocytes (CD4+T cell-depleted) or CD4+ T enriched splenocytes (CD8+ T cell -depleted). In asterisk are the groups considered positive in all mice analyzed based upon the following criteria: spot forming cells (SFC) less 2 standard deviation (SD) was greater than the average of the background plus 2 SD; and the average of the experimental peptides was greater than 10 SFCs per 10^6^ cells. The result shown is the average and standard deviation from two different mice.

In order to see whether the epitope regions identified in the human cohort were capable to elicit immune response in animal model, Balb/c mice were immunized against each immunogenic region and the immune response was assessed (see methods for detail). The Figure 4 depicts level of total specific IgG in different dilution for each peptide either after priming or boosting. The regions 51–65 (Figure 4A), 131–170 (Figure 4C) and 196–210 (Figure 4D) elicited IgG response after both time points analyzed, whereas the region 246–260 (Figure 4E) elicited IgG response only after boosting. The magnitude of production seemed to be as follow: 51–65>131–170>196–210>246–260. The region 71–90 (Figure 4B), was the only one which did not elicit any IgG response in this mice model. IFN-γ production by total splenocytes as well as CD8+ or CD4+ T enriched cells was also evaluated through ELISPOT assay for IFN-γ on immunized mice and controls. Among the regions analyzed epitopes 131–170, 196–210 and 246–260 elicited significant IFN-γ production in total splenocytes. Depletion assays revealed that all T cell response seen was exclusively T CD4+ (Figure 5).

## Discussion

Dengue represents the most important arthropod-borne viral disease in humans in terms of morbidity and mortality and at the present there is no one antiviral therapy available. Although vaccine development is an undisputed public health priority, it remains a considerable scientific challenge due to the four antigenically distinct serotypes and possible risk factors associated to vaccination and disease severity [26]. One possible strategy to avoid pathogeny associated with a dengue vaccine would be to design a vaccine composed of a selected set of epitopes from the four serotypes. The majority of the epitopes involved in dengue neutralization might be present in the E-glycoprotein, which is the major surface protein in the viral particles [14].

Most dengue vaccine candidates and diagnostic methods available nowadays, make use of some part of the E glycoprotein and these peptides might be able to induce cellular and humoral responses. An effective vaccine will need to elicit durable neutralizing-antibody responses, [27] and it is known that mature B-cell memory responses require cytokines from T helper (Th) cells. Synthetic peptides prepared from the sequences of the structural glycoproteins of several viruses have been evaluated for Th cell activity [28]. However, the identification of B-cell epitopes is also important for vaccine design, development of diagnostic reagents and also for studies to elucidate the interactions between virus and antibody on a molecular level [13].

The purpose of this study was to locate linear B-cell epitopes on the dengue virus envelope glycoprotein, which might be potentially useful in diagnosis, vaccine design and immunological studies.

The ELISA results showed that the human serum from patients reacted with eleven continuous peptides, IgG-specific, distributed in five regions at amino acids positions 51–65, 71–90, 131–170, 196–210 and 246–260. Some immunogenic peptides have been identified in the E-glycoprotein of DENV-1 and DENV-2, but not much information is available about B-cell epitopes present in the E-glycoprotein of DENV-3. In 2001 and 2005, some reports described the presence of non specified epitopes in E-glycoprotein of DENV-3 [6], [14].

All the eleven peptides mapped are present in the hydrophilic regions of the protein, suggesting that they could be exposed on the surface of the E-glycoprotein. However, the interface between monomers within a dimer is a mixture of hydrophobic (60%) and hydrophilic (40%) interactions. So, peptides that appear to be hydrophilic based on the prediction tools could not be accessible to the antibody binding. The accessibility and antigenicity predictions showed that from eleven, only three peptides (27, 29 and 30) seems to be exposed at the surface and six (11, 15, 16, 27, 28 and 50) are the most antigenic. The homology modeling of the 3-D structure shown in Figure 2 suggests that peptides 27, 29 and 30 (in blue) are being exposed in the molecule like loops projecting for the surface. The peptide 50 (violet) is the most hidden in the dimer interface but it appears as one of the most antigenic according to the prediction. Peptide 11 (in green), despite being one of the most antigenic does not seem to be the most exposed, according Figure 2. The monomers in the E-glycoprotein make interactions with each other and one of this region contact is on domain II (residues 256–265), which interacts with the same residues across the dyad axis [29]. Peptide 50, spanning residues 246–260, seems to make part of this contact region between monomers.

The results presented demonstrate that was not possible to find the exact location of the carbohydrates in the E-glycoprotein of DENV-3seq, but asparagine (Asn) residues are found in peptides 29, 30 and 31, spanning amino acids 141-165. There are two glycosylated asparagines on each dengue E subunit: Asn-153 on domain I and Asn-67 on domain II. Asn-153 is conserved in most flavivirus envelope proteins and bears a tetrasaccharide, where the fourth sugar (a mannose), appears to be important for viral entry [30].

The E-glycoprotein has three structural domains: DI (spanning residues 130–185), DII (spanning residues 50–130 and 185–300) and DIII (spanning residues 300–400). From the eleven mapped peptides, those included at positions 51–65 (peptide 11), 71–90 (peptides 15 and 16), 196–210 (peptide 40) and 246–260 (peptide 50) are located on DII and those included on positions 131–170 (peptides 27, 28, 29, 30, 31 and 32) are on DI. This latter domain contains predominately type-specific non-neutralizing epitopes. On the other hand, DII contains many cross-reactive epitopes eliciting neutralizing and non-neutralizing monoclonal antibodies that bind to fusion peptides, while DIII contains multiple type and subtype specific epitopes and several conformational virus-neutralizing epitopes [31], [32]. Several additional studies focused on the understanding of the biological properties of these epitopes on protection (neutralization) or pathogenicity (antibody dependent enhancement - ADE) are on going.

The E-glycoprotein is a class II fusion protein, typical of flaviviruses and alphaviruses, where a proteolytic cleavage yields mature virions with the fusion peptide primed for fusion. These fusion peptides are a hydrophobic sequence conserved among all flaviviruses and present on domain II (spanning residues at positions 98–109 for DENV-2), hidden on the dimer interface and becoming exposed during the conformational change initiated by exposure to low pH [6], [30], [33]. The peptides 15–16 are adjacent to the putative DENV3 fusion peptide regions and hence potentially neutralizing. However, mapping to the fusion loop was not always associated with therapeutic activity. Thus, has been demonstrated that antibodies targeting this region could be protective against infection in some cell types and possibly pathological in others [32].

In this work, positive patient sera, with different types of viral infections, and normal individual sera were tested against the eleven mapped peptides, in order to evaluate their ability to discriminate infections between DENV-3 and non-dengue patients and between DENV-3 and infections for other dengue serotypes (DENV-1 and DENV-2). It is important to note that there is no DENV-4 circulation in our region; wherefore a study of a cross-reactive response using sera from patients infected with this serotype was not done due to the lack of available DENV-4 samples.

The results about the ability of the antibodies against the eleven peptides in discriminating the dengue infections showed that specific antibodies against the peptides 15, 16, 28, 29, 30, 31, 32, 40 and 50 can discriminate between DENV-3 and non-dengue infections. These results indicated that these peptide epitopes could be used as antigen in diagnostic kits. The evaluation of the peptide epitopes in discriminating serological responses to DENV-3 from other dengue serotypes (DENV-1 and DENV-2), showed that peptides 11, 27 and 28 were able to differentiate these groups. The reasons that patients previously exposed to DENV 1-2 serotypes produce a stronger antibody titer against these DENV 3 peptides are not clear. Maybe, these peptide regions are more exposed or are more immunogenic in the context of the DENV-1 and DENV-2 E-glycoprotein. In the case of peptides 11 and 27 we found that they are not responsive to DENV-3 when compared to normal individuals (data not shown), so it might have a specific diagnostic potential to DENV 1–2 serotypes. However more studies are required to verify this assumption. On the other hand, peptide 28 showed to be a promiscuous reactive peptide once it reacted well with serum of all dengue patients (DENV-1–2 and 3).

It has been demonstrated that for a number of viruses, Th-cell epitopes can be modeled using synthetic peptides and that active Th-cell epitopes can modulate the antibody response from B-cell epitopes. These observations are important in vaccine design and mean that effective vaccines will most likely require co-expression of B- and T-cell epitopes [34]. However, the results showed here that peptide 27 despite of appearing in all predictions and curiously also being a putative CD4+ T-cell epitope [35], does not differentiate well between dengue 3 and non-dengue patient serum but can be a potential candidate as a marker for serotype diagnostic.

For further characterization of the DENV3 envelope regions recognized by sera of naturally dengue-infected people, Balb/c mice were immunized with each epitope and the humoral and cellular immune responses were assessed. Four out of 5 dengue epitopes identified in this report showed to be immunogenic in Balb/c mice. The region 51–65 induced the strongest antibody response despite no IFN-γ response was detected in mice immunized with this epitope, suggesting that this immunization might be eliciting humoral immune response in a T cell independent manner. The envelope epitopes 131–170, 196–210 and 246–260 elicited both IgG production and T CD4-restricted response. The epitope 71–90 did not induce neither humoral nor IFN-γ response in this mice model. It's quite interesting that such small peptides (15-mer) can elicit not only T cell, but also B cell response as shown in this report. Reynolds et al [36] recently using an experimental autoimmune glomerulonephritis (EAG) model have shown that WKY rat immunized with the 15mer pcol (24–38) - (FTRHSQTTANPSCPE), a stretch of a human B cell epitope in the N terminal of the alpha 3 chain of type IV collagen; elicited either strong B cell or T cell response. Thus, we believe that as long as the 15-mer encompasses a B cell epitope, it can elicit both B and T cell responses. Corroborating with that, Singh et al [37] reported that a peptide sequence as small as 12-mer long could prime for antibody, T cell proliferative as well as for DHT response in different mice strain, including Balb/c. Thus, the antibodies produced against these 15mers in Balb/c can be used to further analyze some properties of these anti-serums, such as virus neutralizing activity.

Our study identified several immunodominant IgG-specific epitopes on the envelope of DENV-3. The peptides of the E glycoprotein described herein in conjunction with other well-documented epitopes are potentially relevant for the development of diagnostic reagents and a proposal to an effective vaccine for the dengue virus.
