# Correction: Identification of Continuous Human B-Cell Epitopes in the Envelope Glycoprotein of Dengue Virus Type 3 (DENV-3)

**DOI:** 10.1371/annotation/238cbff8-6794-4796-8a8a-a80d3b246757

**Published:** 2009-10-27

**Authors:** Andréa N. M. Rangel da Silva, Eduardo J. M. Nascimento, Marli Tenório Cordeiro, Laura H. V. G. Gil, Frederico G. C. Abath, Silvia M. L. Montenegro, Ernesto T. A. Marques

The affiliations for the fourth author are incorrect. Laura H. V. G. Gil is affiliated only with #1 Virology and Experimental Therapy Laboratory, Aggeu Magalhães Research Center, Fiocruz, Recife, Pernambuco, Brazil. 

